# Autophagy-dependent apoptosis is triggered by a semi-synthetic [6]-gingerol analogue in triple negative breast cancer cells

**DOI:** 10.18632/oncotarget.25704

**Published:** 2018-07-20

**Authors:** Liany Luna-Dulcey, Rebeka Tomasin, Marina A. Naves, James A. da Silva, Marcia R. Cominetti

**Affiliations:** ^1^ Laboratory of Biology of Aging, Department of Gerontology, Federal University of São Carlos, CEP 13565-905, São Carlos, SP, Brazil; ^2^ Department of Pharmacy, Federal University of Sergipe, CEP 49400-000, São José, Lagarto, SE, Brazil

**Keywords:** autophagy, breast cancer, caspase-independent apoptosis, cytotoxicity, natural products

## Abstract

Triple negative breast cancer (TNBC) is very aggressive and lacks specific therapeutic targets, having limited treatment options and poor prognosis. [6]-gingerol is the most abundant and studied compound in ginger, presenting diverse biological properties such as antitumor activity against several types of cancer, including breast cancer. In this study, we show that the semi-synthetic analogue SSi6, generated after chemical modification of the [6]-gingerol molecule, using acetone-2,4-dinitrophenylhydrazone (2,4-DNPH) reagent, enhanced selective cytotoxic effects on MDA-MB-231 cells. Remarkably, unlike the original [6]-gingerol molecule, SSi6 enabled autophagy followed by caspase-independent apoptosis in tumor cells. We found a time-dependent association between SSi6-induced oxidative stress, autophagy and apoptosis. Initial SSi6-induced reactive oxygen species (ROS) accumulation (1h) led to autophagy activation (2-6h), which was followed by caspase-independent apoptosis (14h) in TNBC cells. Additionally, our data showed that SSi6 induction of ROS plays a key role in the promotion of autophagy and apoptosis. In order to investigate whether the observed cell death induction was dependent on preceding autophagy in MDA-MB-231 cells, we used siRNA to knock down LC3B prior to SSi6 treatment. Our data show that LC3B downregulation decreased the number of apoptotic cells after treatment with SSi6, indicating that autophagy is a key initial step on SSi6-induced caspase-independent apoptosis. Overall, the results of this study show that structural modifications of natural compounds can be an interesting strategy for developing antitumor drugs, with distinct mechanisms of actions, which could possibly be used against triple negative breast cancer cells that are resistant to canonical apoptosis-inducing drugs.

## INTRODUCTION

Programed cell death (PCD) is an active process that includes a series of orchestrated signals that ultimately lead to cell death [1]. PCD can be further classified into several types; the two main ones being apoptosis and autophagy. Moreover, apoptosis can be dependent or independent on caspases and has been widely studied as a cellular response to DNA damage. However, recent studies suggest that autophagy also plays an important role in determining the final fate of the cell [2]. Although there has been extensive research on caspase-mediated cell death, much less is known about the molecular mechanisms involved in caspase-independent cell death. Caspase-independent apoptosis (CIA) is mediated by several effectors, including the apoptosis-inducing factor (AIF) [3, 4]. Upon reactive oxygen species (ROS) accumulation and apoptotic stimuli, AIF is released from the mitochondria and translocated to the nucleus, where it triggers large-scale DNA fragmentation and nuclear chromatin condensation, leading to CIA [5].

Macroautophagy (autophagy hereafter) refers to the lysosomal degradation mechanism, which is in normal conditions, essential for survival, development, differentiation and cell homeostasis [6, 7]. It is well known that autophagy plays a key role in a variety of cellular processes, such as oxidative stress, metabolism and cell death/survival [8]. However, the role of autophagy in cancer remains controversial and is yet to be elucidated [9]. It has been described that in the early stages, autophagy is an important anti-cancer mechanism preventing cancer initiation [10]. On the other hand, it is believed that autophagy can support cancer progression via its pro-survival action in cells [11].

Autophagy is tightly regulated by a network of upstream signaling cascades [12]. A key protein for this process is light chain 3 (LC3B), which is required for autophagosome assembling. It is commonly used to estimate the abundance of autophagosomes, and therefore, autophagy in the cells [13, 14]. Even though autophagy cell death is different from apoptosis, the complex link between them has been investigated, but is still not well understood [15]. Emerging studies report that autophagy and apoptosis can overlap in cancer cells in response to treatment [16, 17].

Reactive oxygen species (ROS) are free radicals derived from oxygen metabolism [18]. ROS is known to play important roles in various biochemical processes, including apoptosis and autophagy. An excess of ROS, known as oxidative stress, can damage some organelles, which can activate cell death from both tumor and normal cells [19]. Several anti-cancer drugs have been shown to activate ROS-mediated autophagy, which in turn leads to cytoprotective regulation, as well as to activate apoptosis or both [20, 21].

Triple-negative breast cancer (TNBC), which comprises 15% to 20% of all cases, is the most aggressive, and consequently, the one with the worst prognosis amongst all breast cancer subtypes. TNBC lacks expression of both estrogen and progesterone receptors (ER and PR, respectively), as well as lacks or have very low levels of human epidermal growth factor receptor 2 (HER2) [22]. Treatment for TNBC often comprises high doses of chemotherapeutic drugs and radiation. However, these options have limited efficacy and undesired side effects. In addition, tumor cells can develop resistance to therapies; therefore, selective compounds that induce PCD in cancer cells are highly nedeed.

Ginger (*Zingiber officinale* Roscoe) is plant historically used in complementary and alternative medicine [23]. [6]-gingerol (6G) was identified as the major phenolic compound of the rhizomes of the plant. It has been described that 6G has several pharmacological effects, including antitumor activity [24]. This work investigated the effects of SSi6, a semi-synthetic substance produced by chemical modification of 6G [25], on the induction of cell death in MDA-MB-231 cells.

## RESULTS

### Cytotoxicity of SSi6, 6G and acetone-2,4-DNPH

The semisynthetic substance SSi6 (Supplementary Figure 1A) was produced by chemical modification of [6]-gingerol (6G) (Supplementary Figure 1B), using the organic compound acetone-2,4-dinitrophenylhydrazine (2,4-DNPH) (Supplementary Figure 1C). Treatment of MDA-MB-231 or MCF-10A cells with SSi6 induced morphological changes; however, this effect was evident much earlier and more prominently in tumor cells, which at 2h of incubation with 50μM and over, acquired a round shape, accompanied by a loss of density (Supplementary Figure 2A). At 48h of treatment, SSi6 induced dramatic morphological changes in MDA-MB-231 cells at concentrations starting from 25μM. At this point, a total lack of adherence and the presence of cellular debris were observed, indicating cell death. On the other hand, only mild changes were observed in non-malignant cells (MCF-10A) incubated with the highest concentrations (50 and 100μM) of SSi6 and in the longest incubation time (48h) (Supplementary Figure 2B). In addition, the activity of SSi6 was tested in non-TNBC cells such as MCF-7 (ER receptor) and SKBR3 (HER2 receptor). As observed in Supplementary Figure 3, SSi6 does not induce the formation of cytoplasmic vacuoles in these cells.

Cytotoxicity against MDA-MB-231, MCF-10A, MCF-7 and SKBR3 cells was evaluated and the results expressed as IC_50_ values are listed in Table 1. SSi6 exhibited a slightly greater cytotoxicity (IC_50_ 22.90±0.35μM) against MDA-MB-231 in comparison to MCF-10A (IC_50_ 34.17±2.49μM), with a selectivity index of ~1.49 for malignant cells after 48h of treatment. On the other hand, 6G exhibited IC_50_ values of 404.5±7.6μM for MDA-MB-231, 985.8±0.57μM for MCF-7, 316.2±0.61μM for SKBR3 and 599.4±8.5μM for MCF-10A cells, while 2,4-DNPH presented IC_50_ values >100μM for all cell lines, thus both 6G and 2,4-DNPH were much less active than its semi-synthetic counterpart SSi6. Therefore, according to the presented results, we demonstrated that the chemical modification performed in 6G improved approximately 17 times in the IC_50_ value for cytotoxicity on TNBC cells. In non-TNBC cells, MCF-7 and SKBR3 SSi6 cytotoxicity was lower compared to TNBC cells (Table 1). As shown in Supplementary Figure 3 the effects on the morphology of these same cells treated with SSi6. According to these results, SSi6 presents higher cytotoxic activity in MDA-MB-231 cells; therefore, the mechanisms of death presented hereafter will be performed in TNBC cells. The human primary dermal fibroblast (HPDF) cell line was also used to investigate SSi6 cytotoxicity (Supplementary Figure 4), showing once again that SSi6 has low cytotoxicity against non-tumor cells.

**Table 1:** IC_50_ values of 48h treatment of [6]-gingerol (6G), 2,4-DNPH and SSi6 in the MDA-MB-231, MCF-10A, MCF-7 and SKBR3 cell lines

Substances	IC_50_ ±SD(μM)				
	MDA-MB-231	MCF-10A	MCF-7	SKBR3	SI^a^
**6G**	404.5±17.6	599.4±18.5	985.8±0.57	316.2±0.61	1.48
**2,4-DNPH**	>100	>100	>100	>100	------
**SSi6**	22.90±0.35	34.17±2.49	31.01±3.2	21.93±0.67	1.49

Values are expressed as mean ± SD of three independent assays in triplicate

SD = Standard deviation

^a^(SI) Selectivity index: IC_50_ MCF-10A/IC_50_ MDA-MB-231.

**Figure 1:**
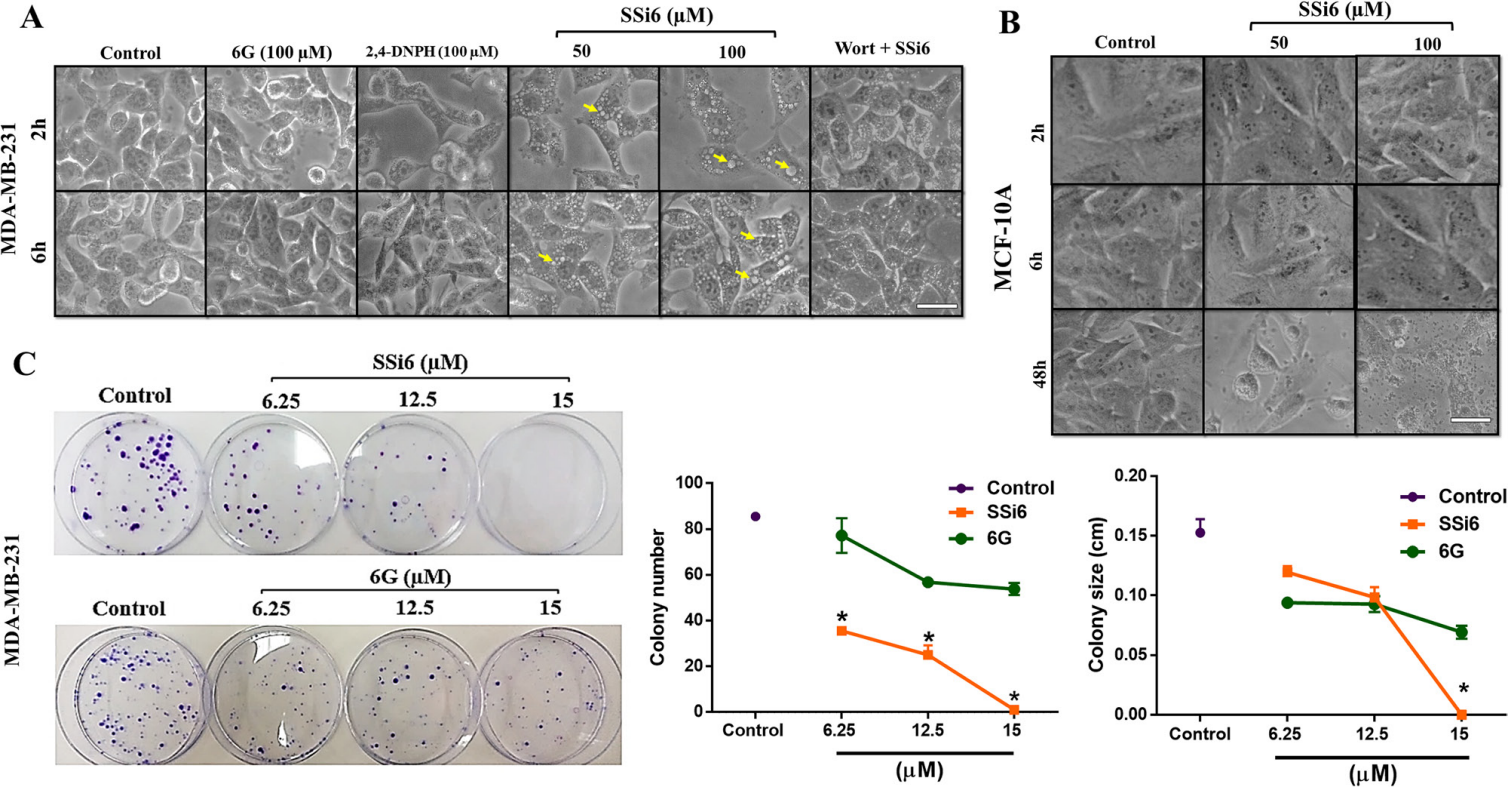
Effects of SSi6 and 6G on morphology and colony formation of triple negative breast cancer MDA-MB-231 cells **(A)** Detection of cytoplasmic vacuoles in MDA-MB-231 after incubation with SSi6 for 2 and 6h (yellow arrows). Cells were examined using an inverted microscope (amplification 200×), scale bar = 50μm. **(B)** As a control, non-tumorMCF-10A cells treated (2, 6 and 48h) with SSi6 at the same conditions did not present cytoplasmic vacuoles, even at high concentrations and longer incubation time (48h). **(C)** Clonogenic assay of MDA-MB-231 cells treated with indicated concentrations of SSi6 and 6G for 48h. A photography of Petri-dishes from a representative experiment is shown with graph quantifications of colony number (^*^p<0.0001) and size (^*^p<0.05). Data represent mean ± SD of three independent tests in triplicate. Results were compared using ANOVA, followed by a Tukey's post-hoc analysis.

Interestingly, the presence of cytoplasmic vacuoles in MDA-MB-231 cells subjected to SSi6 treatment (50 and 100μM) was evident from 2 and 6h of incubation (Figure 1A), which was not observed in non-malignant MCF-10A in the same times of treatment after 48h (Figure 1B). Neither 6G nor 2,4-DNPH evoked any changes in the cell morphology, even at the highest concentration and longest incubation time point. Wortmannin, an indirect autophagy inhibitor [26, 27], was able to completely abrogate vacuole formation (Figure 1A), indicating that SSi6-induced cytoplasmic vacuoles are probably linked to the occurrence of autophagy in these cells.

Clonogenic assay is an *in vitro* cell survival experiment based on the ability of a single cell to grow into a colony, and it is used to evaluate the reproductive capacity of cells after exposure to cytotoxic agents [28]. When compared to negative control (DMSO 1%), SSi6 at 6.25, 12.5 and 15μM significantly reduced the number and size of TNBC colonies in a concentration-dependent fashion. The highest concentration (15μM) was sufficient to completely impair colony formation. In contrast, when cells were treated with 6G there was no significant inhibition in either the number or in the size of MDA-MB-231 colonies (Figure 1C).

### SSi6 induces ROS generation, autophagy and caspase-independent apoptosis

**Figure 2:**
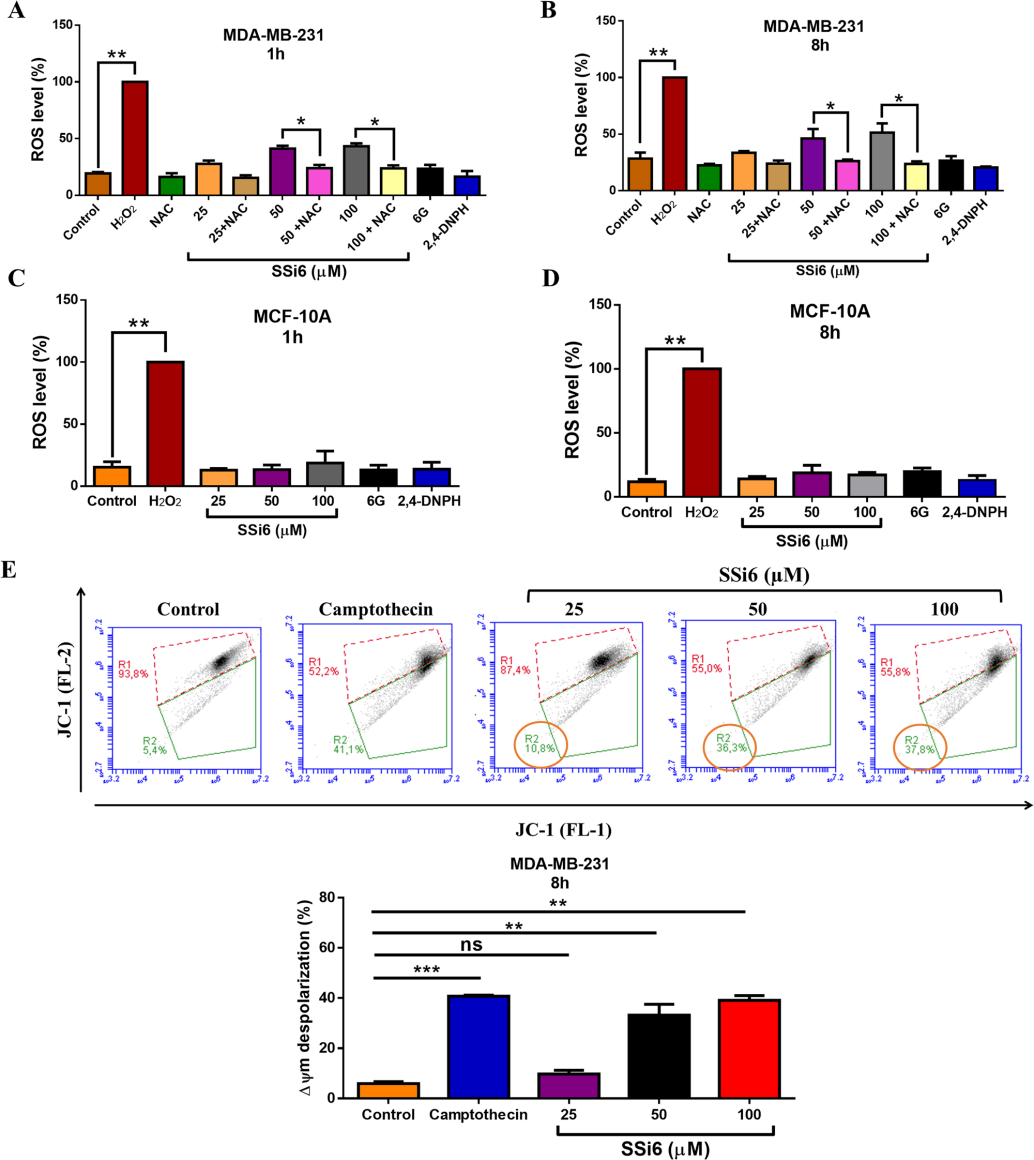
Effects of SSi6 in ROS formation and mitochondrial membrane potential MDA-MB-231 cells were treated with indicated concentrations of SSi6, 6G and 2,4-DNPH with or without NAC (5mM) for **(A)** 1 and **(B)** 8h followed by incubation with H_2_DCFDA. MCF-10A cells were treated with SSi6, 6G and 2,4-DNPH for **(C)** 1 and **(D)** 8h and analyzed by fluorescence. Data are presented as mean ± SD (n=3 in each group). ^*^p<0.01, ^**^p<0.001 *vs* control group using ANOVA followed by a Tukey's post-hoc analysis. **(E)** Loss of mitochondrial membrane potential (Δψm) induced by SSi6 is concentration and time-dependent. Δψm depolarization was monitored by flow cytometric analysis of JC-1 mitochondrial potential marker. MDA-MB-231 cells treated with indicated concentrations of SSi6 for 8h and stained with JC-1 as described in material and methods. Camptothecin (200μM) was used as positive control (apoptosis induction agent). Gated region R1 (red) includes cells with intact mitochondrial membranes and gated region R2 (green) depicts cells with loss of Δψm. Graphic representation of mean values for R2 region data (cells with Δψm collapse). Data represents mean ± SD of three independent assays in triplicate. Significance at the ^**^p<0.001, ^***^p<0.0001 level using ANOVA followed by a Tukey's post-hoc analysis.

In order to elucidate the underlying mechanisms involved in SSi6-induced cell death, we estimated the changes in the ROS levels in SSi6, 6G and 2,4-DNPH-treated cells using a H_2_DCFDA reagent. As shown in Figure 2A and 2B, there was a significant increase in the fluorescent content in MDA-MB-231 cells after 1 and 8 hours of SSi6 treatment, reflecting the accumulation of ROS in these cells. On the other hand, 6G and 2,4-DNPH did not cause generation of ROS in TBNC cells. *N*-acetylcysteine (NAC) is an antioxidant that replenishes intracellular glutathione (GSH) protecting the cells from oxidative stress [29]. We used the antioxidant NAC to further confirm whether SSi6 treatment was responsible for the increase in ROS. Co-treatment of NAC significantly blocked the generation of ROS in both treatment times (1 and 8h). In MCF-10A cells, ROS levels were not significant after the treatment with SSi6, 6G and 2,4-DNPH, which explains the low cytotoxic effects of SSi6 on non-tumor cells (Figure 2C and 2D). It was also demonstrated that SSi6-induced ROS production resulted in a partial mitochondrial membrane depolarization (Figure 2E).

**Figure 3:**
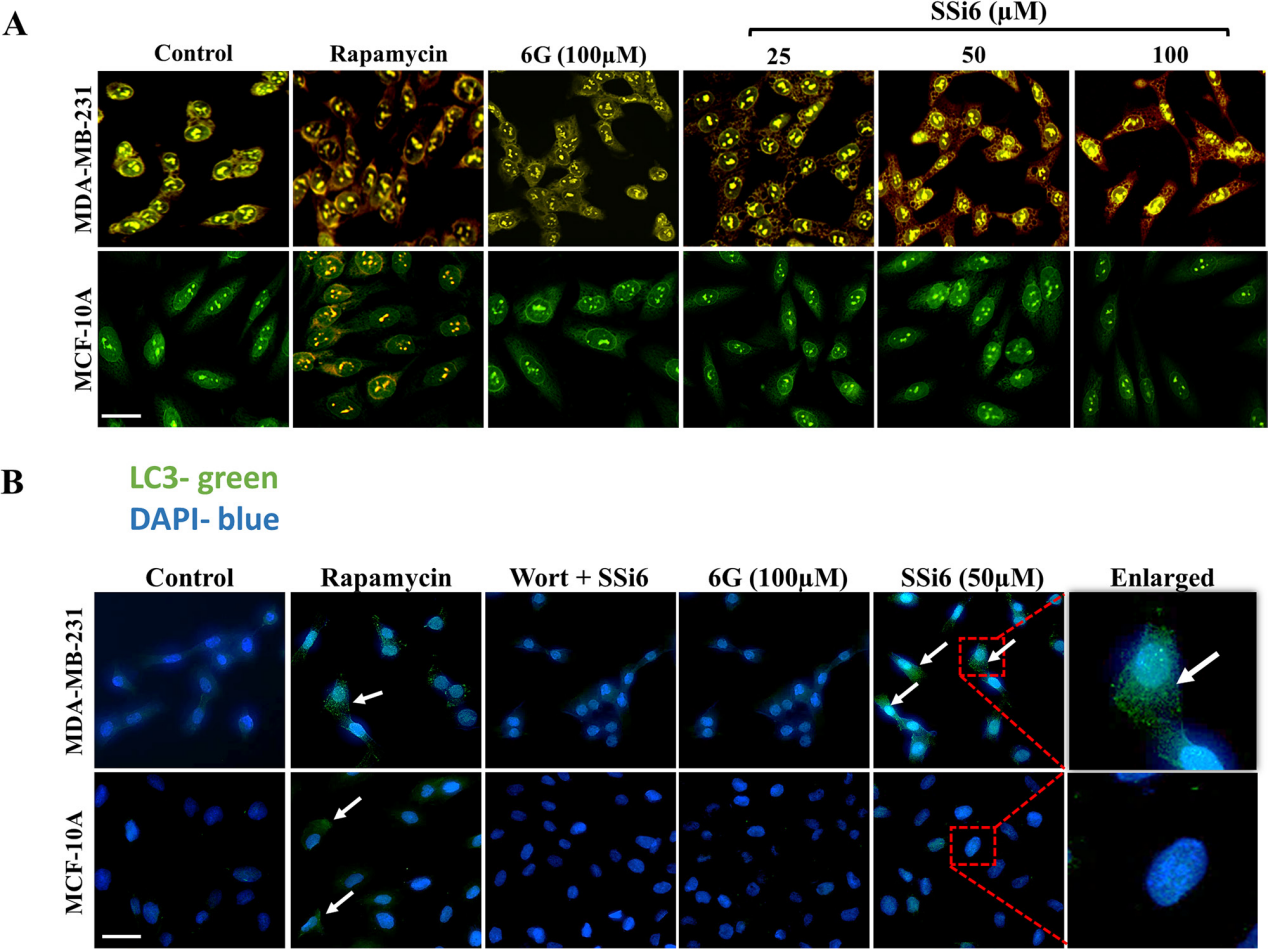
Effects of SSi6 and 6G on induction of autophagy **(A)** MDA-MB-231 and MCF-10A cells were treated with indicated concentrations of SSi6 and 6G for 6h. Rapamycin 500nM for 24h was used as a positive control of autophagy. Cells were fixed, stained with acridine orange (AO) and images were captured with ImageXpress micro, detecting the formation of acid vesicular organelles (AVOs). Nuclei were stained with DAPI, scale bar = 50 μm. **(B)** MDA-MB-231 and MCF-10A were treated with SSi6 (50μM) and 6G (100μM) for 6h. Cells were incubated with anti-LC3B and Alexa fluor 488^®^. Wortmannin was used as an inhibitor of autophagy, previously to the treatment with SSi6 (Wort 30μM + 50μM of SSi6). Images were obtained with amplification of 400×. White arrows indicate the labeling of the LC3B protein after incubation of rapamycin and SSi6 for 24 and 6h, respectively.

To confirm the nature of the cytoplasmic vacuoles previously observed in the morphology assay, a test for acid vesicular organelles (AVOs) detection was carried out using acridine orange [30]. Rapamycin (500nM), an inductor of autophagy, was used as a positive control (24h incubation). SSi6 induced, in a concentration-dependent manner, the occurrence of AVOs in MDA-MB-231 cells after 6h of treatment, shown by a strong red fluorescence in the cytoplasm, which was absent in non-malignant MCF-10A cells (Figure 3A). In addition, an immunostaining assay was performed to detect LC3B, a protein that is considered an autophagy marker [12, 31]. Similarly, the induction of autophagy was clear after 6h of SSi6 (50μM) treatment in MDA-MB-231 cells but was absent in non-malignant MCF-10A cells (Figure 3B). In contrast, 6G (100μM) did not evoke LC3B expression in malignant or in non-malignant cells. When wortmannin, an indirect autophagy inhibition control [27], was previously incubated with SSi6 (30μM wortmannin + 50μM SSi6) there was a block in the synthesis of LC3B protein (Figure 3B). Overall, these data indicate that SSi6 is a potent inducer of autophagy in TNBC cells, after relatively short incubation times (2-6h). It is important to mention that SSi6 leads to vacuole formation only up to 6 hours of treatment. After that, changes in cell morphology and density can be observed in 14-24h of treatment.

**Figure 4:**
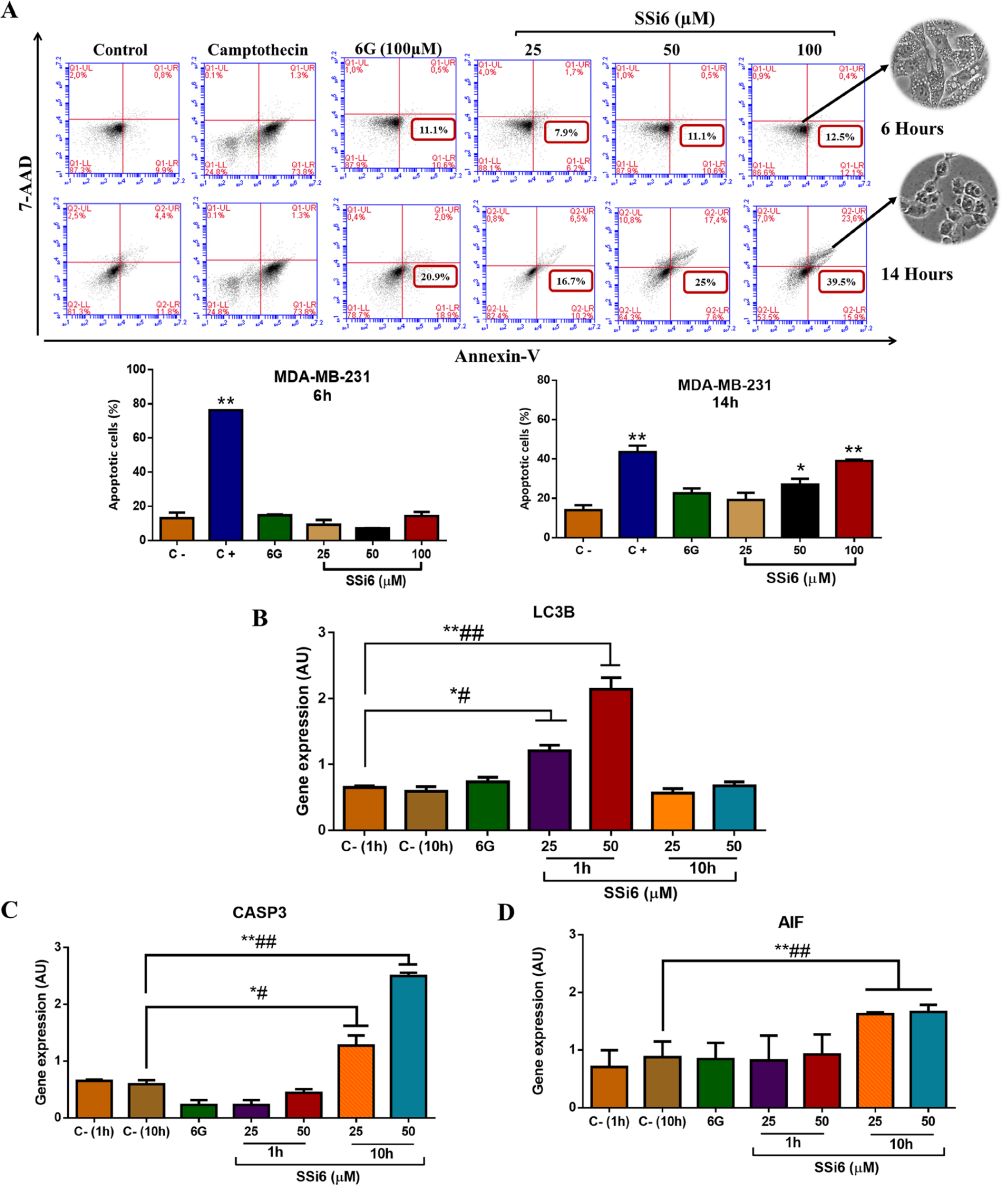
Effects of SSi6 and 6G in apoptosis and expression of apoptosis- and autophagy-related genes in TNBC cells **(A)** Cytometry analysis of MDA-MB-231 cells treated with indicated concentrations of SSi6 and 6G for 6 and 14h, respectively. Camptothecin (200μM) was used as positive control of apoptosis (treatment for 24h). Data represents mean ± SD of three independent assays in triplicate. Significance at the ^*^p<0.001, ^**^p<0.0001 level using ANOVA followed by a Tukey's post-hoc analysis. **(B)** Cells were incubated with indicated concentrations of SSi6 and 6G for two different times and in independent assays: 1h-6G, 1h-SSi6 and 10h-SSi6 to determine LC3B gene expression and **(C)** 10h-6G, 1h-SSi6 and 10h-SSi6 to evaluate expression of the CASP3 and **(D)** AIF genes. Negative control (C-) represents cells with no treatment. Total RNA was extracted and cDNAs were synthesized. Amplification of endogen control (Rpl37a) and each of the target genes was performed using real-time PCR equipment. Data represent mean ± SD of three independent assays in triplicate. Results were compared using ANOVA, followed by a Tukey's post-hoc analysis. Significance at the ^*^p<0.05, ^**^p<0.001, #*p*<0.01, ##*p*<0.001 *vs* negative control in the respective time.

To further characterize the type of cell death involved in the cytotoxic effects observed in MDA-MB-231, cells were incubated with SSi6 or 6G for 6 or 14h, stained with PE-Annexin-V and 7-AAD, and analyzed by flow cytometry (Figure 4A). At 6h of treatment, there was no significant occurrence of apoptosis in either 6G or SSi6-treated MDA-MB-231 cells compared to untreated controls. On the other hand, after 14h of treatment with SSi6 (100μM), there was a remarkable increase in the percentage of apoptotic cells, indicating that the initial events of autophagy (2-6h) were followed by apoptotic cell death (14h) upon incubation with SSi6. After 14h of incubation with SSi6 at 100μM the percentage of TNBC cells in apoptosis was 39.5%, while after the same time and concentration, 6G induced 20.9% of apoptosis. In fact, this difference does not appear to be very significant, however if we analyze the effects on autophagy, as demonstrated by cellular morphology with the presence of high amounts of vacuoles and by the formation of acid vesicles, SSi6 was much more effective inducing autophagy compared to 6G. In this sense, it seems that the most important effect of adding 2,4-DNPH to 6G is the induction of autophagy.

mRNA and protein expression levels of autophagy and apoptosis-related genes were accessed in SSi6-treated TNBC cells. LC3B mRNA was upregulated after 1h of incubation with SSi6; however, the levels returned to normal after 10h of incubation (Figure 4B). On the other hand, after 1h of treatment with SSi6 there was no positive CASP-3 gene regulation. Nevertheless, after 10h this gene had its expression positively regulated compared to the control (Figure 4C). Regarding the AIF mRNA expression, while no significant changes were observed after 1h SSi6 incubation, there was a significant upregulation at 10h of treatment (Figure 4D). Neither of these alterations were observed when the treatments were conducted using 6G, which is in agreement with our previous assays indicating that 6G did not induce autophagy and had only mild apoptotic effects in TNBC cells at these conditions.

**Figure 5:**
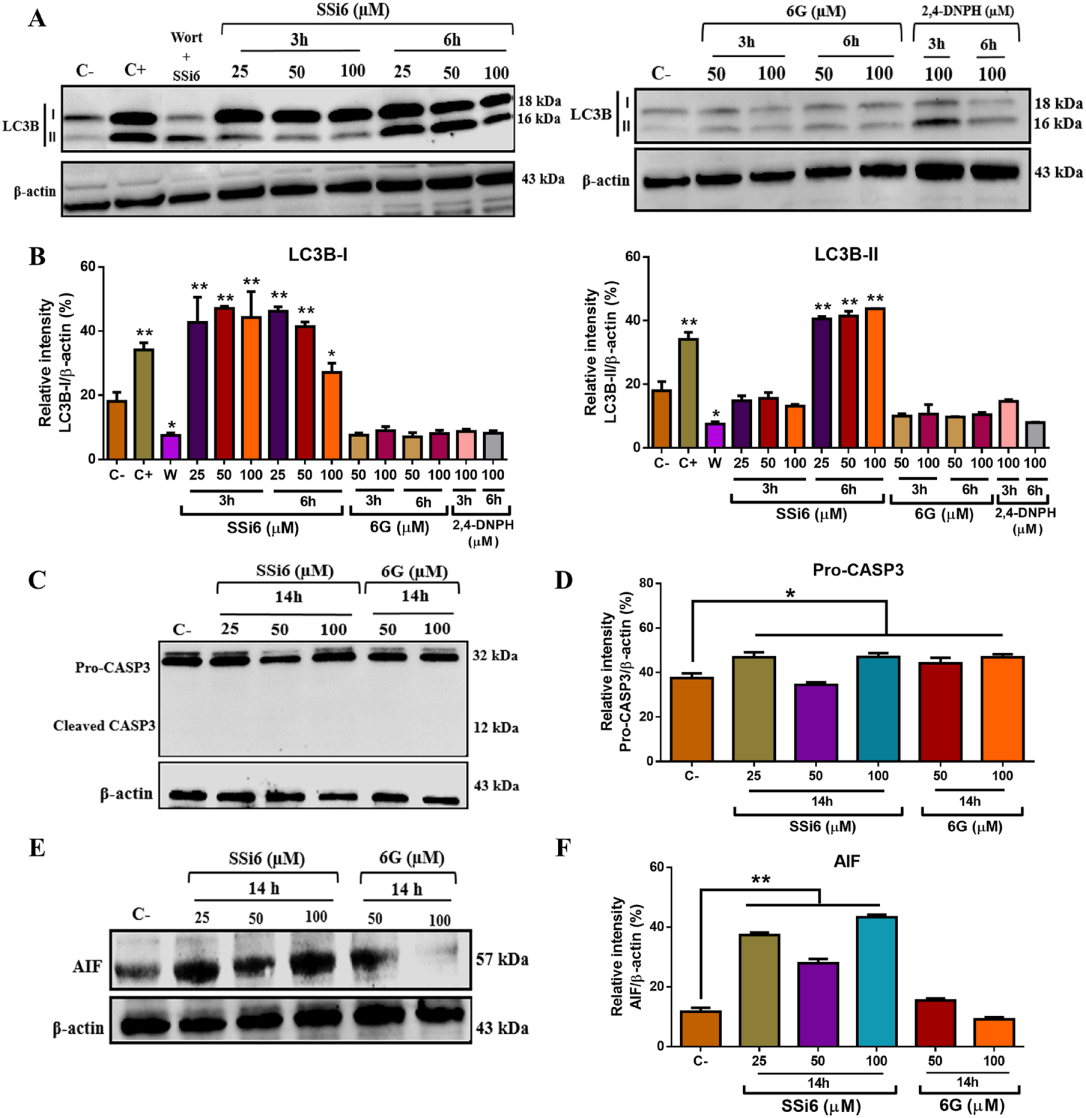
Effects of SSi6, 6G and 2,4-DNPH on the levels of apoptosis- and autophagy-related proteins in TNBC cells **(A)** MDA-MB-231 cells were treated with indicated concentrations of SSi6, 6G and 2,4-DNPH for 3 and 6h. Cells were then harvested and subjected to western blot analysis of LC3B (LC3B-I and LC3B-II) protein. **(B)** Protein expression was quantified using ImageJ software, means ± SD were presented (^*^p<0.05; ^**^p<0.01, Tukey's post-hoc analysis). **(C)** MDA-MB-231 cells were harvested after treatment with indicated concentrations of SSi6 and 6G for the indicated period of time, the western blot was then performed for the detection of caspase-3 (pro-casp3 and cleaved casp3). β-actin was used a endogen control. **(D)** Protein expression was quantified using ImageJ software, means ± SD were presented (^*^p<0.05, Tukey's post-hoc analysis). **(E)** Cells were treated with indicated concentrations of SSi6 and 6G for 14h, followed by western blot to detect AIF (caspase-independent apoptosis marker). **(F)** Protein expression was quantified using ImageJ software, means ± SD were presented (^**^p<0.001, Tukey's post-hoc analysis). The controls performed corresponded to the C- (negative control-DMSO 1%), C+ (positive control of autophagy induction-rapamycin 500nM-24h) and Wort + SSi6 (control of autophagy inhibition - 30μM wortmannin + 100μM SSi6-6h).

We have also examined changes in LC3B protein levels upon SSi6, 6G and 2,4-DNPH treatment. Conversion of LC3B-I into its lipidated form at the C-terminal end (LC3B-II) is regarded as a key marker of autophagy [32]. SSi6 markedly increased the lipidation of LC3B (LC3B-II) in a concentration and time-dependent manner in MDA-MB-231 cells (Figure 5A and 5B). The co-treatment of TNBC with SSi6 (100μM) and wortmannin (30μM) abolished the induction of autophagy, as demonstrated by the reduced levels of LC3B-II, which corroborates the results found in RT-qPCR, again indicating the occurrence of autophagy. As expected, 6G and 2,4-DNPH had no effect on LC3B levels in TNBC cells (Figure 5A and 5B).

Once our data indicated that apoptosis is a later event in SSi6-induced toxicity, AIF and caspase-3 protein levels were accessed after 14h of treatment, however no cleaved caspase-3 expression was detected (Figure 5C and 5D). An important point is that the measurement of caspase-3 was performed in 19, 24 and 28 hours of treatment, showing the same result in all analyzed times (data not shown). In the other hand, AIF levels increased upon treatment, especially with SSi6 (Figure 5E and 5F), indicating the involvement of a caspase-independent mechanism on apoptosis induction by SSi6 in TNBC cells.

### SSi6 inhibits autophagic flux in MDA-MB-231 cells

**Figure 6:**
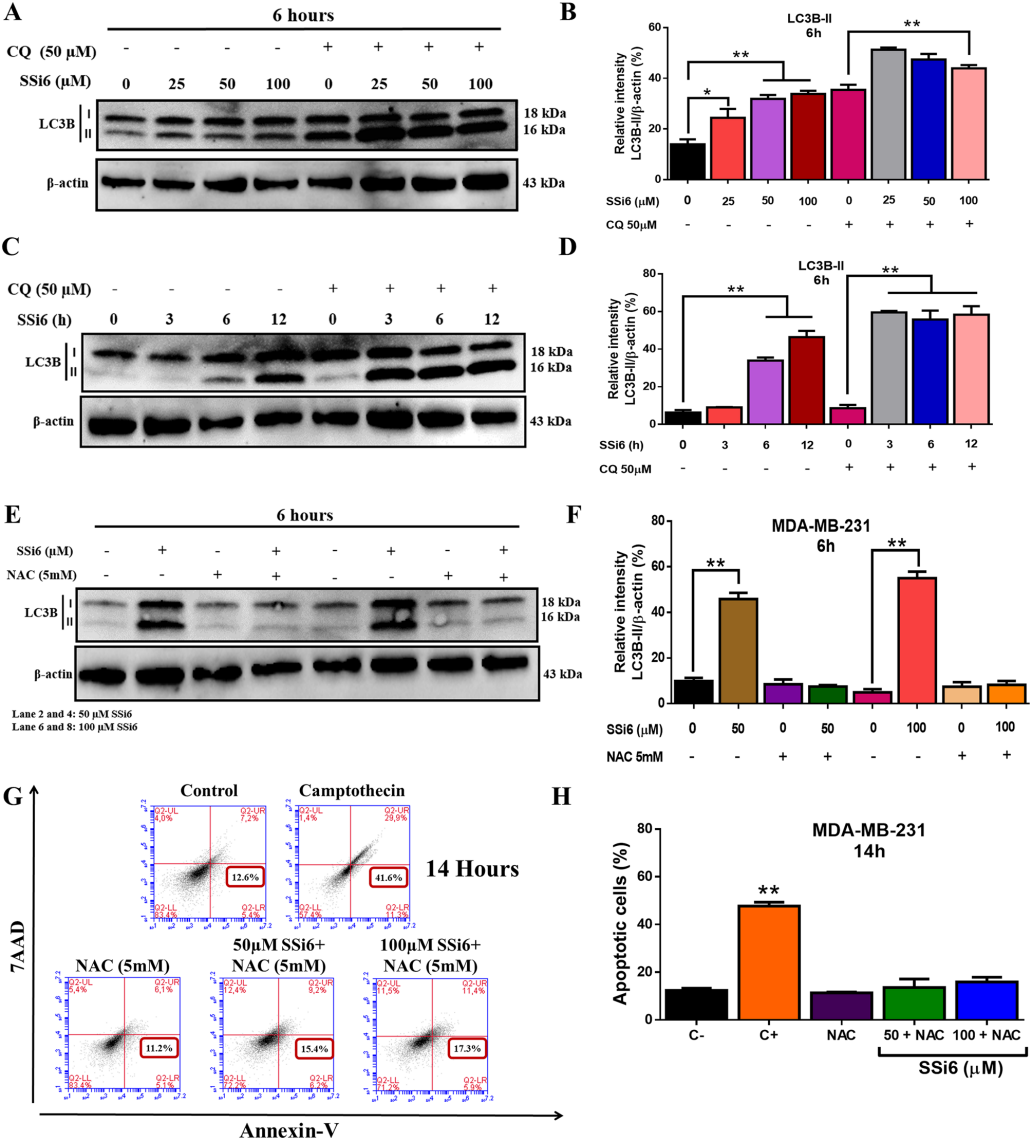
SSi6 increases autophagic flux and induces autophagy and apoptosis via the activation ROS production **(A)** MDA-MB-231 cells were treated with the indicated concentrations of SSi6 with or without chloroquine (CQ, 50μM) for 6h. Cell lysed were collected to western blotting for detecting LC3 conversion. **(B)** LC3B-II expression was evaluated by ImageJ software, means ± SD were presented (^*^p<0.01; ^**^p<0.001, Tukey's post-hoc analysis). **(C)** Cells were treated with SSi6 (100μM) for the indicated time points with or without CQ (50μM). **(D)** Protein levels (LC3B-II) was analyzed by ImageJ software, means ± SD were presented (^**^p<0.0001, Tukey's post-hoc analysis). β-actin was used as a loading control. **(E)** MDA-MB-231 cells were treated with SSi6 (50 and 100μM) with or without NAC (5mM) for 6h. Cells lysates were analyzed by western blotting for detecting LC3B-II. β-actin was used as endogen control. **(F)** The levels of LC3B-II were analyzed by ImageJ software, means ± SD were presented (^**^p<0.0001, Tukey's post-hoc analysis). **(G)** Cells were treated with SSi6 (50 and 100μM) for 7h, then treated with NAC (5mM) for additional 7h, and analyzed by cytometry using 7AAD and Annexin-V. Camptothecin (200μM) was used as positive control of apoptosis. **(H)** The apoptotic rate was determined by fluorescence intensity using flow cytometry. Results are presented as mean ±SD from three independent experiments (^**^p<0.001 *vs* control group, Tukey's post-hoc analysis).

Lysosomal degradation of autophagosomes leads to a decrease in LC3B-II levels during autophagy [32]. Blockage of autophagic flux correlates with increased levels of LC3B-II, which contributes to cell death [33, 34]. Our data showed that SSi6 markedly increased the lipidation of LC3 (LC3B-II) in a concentration-dependent manner (Figure 6A and 6B). In addition, the blockage of autophagosomal degradation induced by chloroquine (CQ), an indirect autophagy inhibitor, caused time-dependent (Figure 6C and 6D) LC3B-II accumulation, indicating a decrease in autophagic flux [27, 32].

### Role of ROS in the promotion of autophagy and apoptosis by SSi6

Considering that ROS are involved in the induction of both processes (autophagy and apoptosis) [35, 36], it was necessary to investigate whether ROS blockade would affect the activation of these cell death processes. To determine how much of the total autophagy rate was linked to ROS; MDA-MB-231 cells were co-treated with NAC (5mM) for 6h. Western blotting analysis showed that there was a significant reduction in the level of LC3B isoforms (Figure 6E and 6F). Next, the role of ROS generation in SSi6-induced apoptosis was investigated. In this case, the NAC (5mM) was added only after 7h to the cells to not affect the initial induction of autophagy, subsequently the apoptotic rate was measured at 14h by flow cytometry. After this time, the apoptotic rate decreased significantly, evidencing as in the previous case, the connection of this process with ROS (Figure 6G and 6H). In SSi6-treated MDA-MB-231 cells, the apoptotic rate, at the same incubation time and at 100μM, was 39.5% (see Figure 4A). Taken together, the results indicate that ROS generation by SSi6 participates in the induction of autophagy and apoptosis.

### LC3B knockdown attenuates SSi6 apoptotic effects in TNBC cells

**Figure 7:**
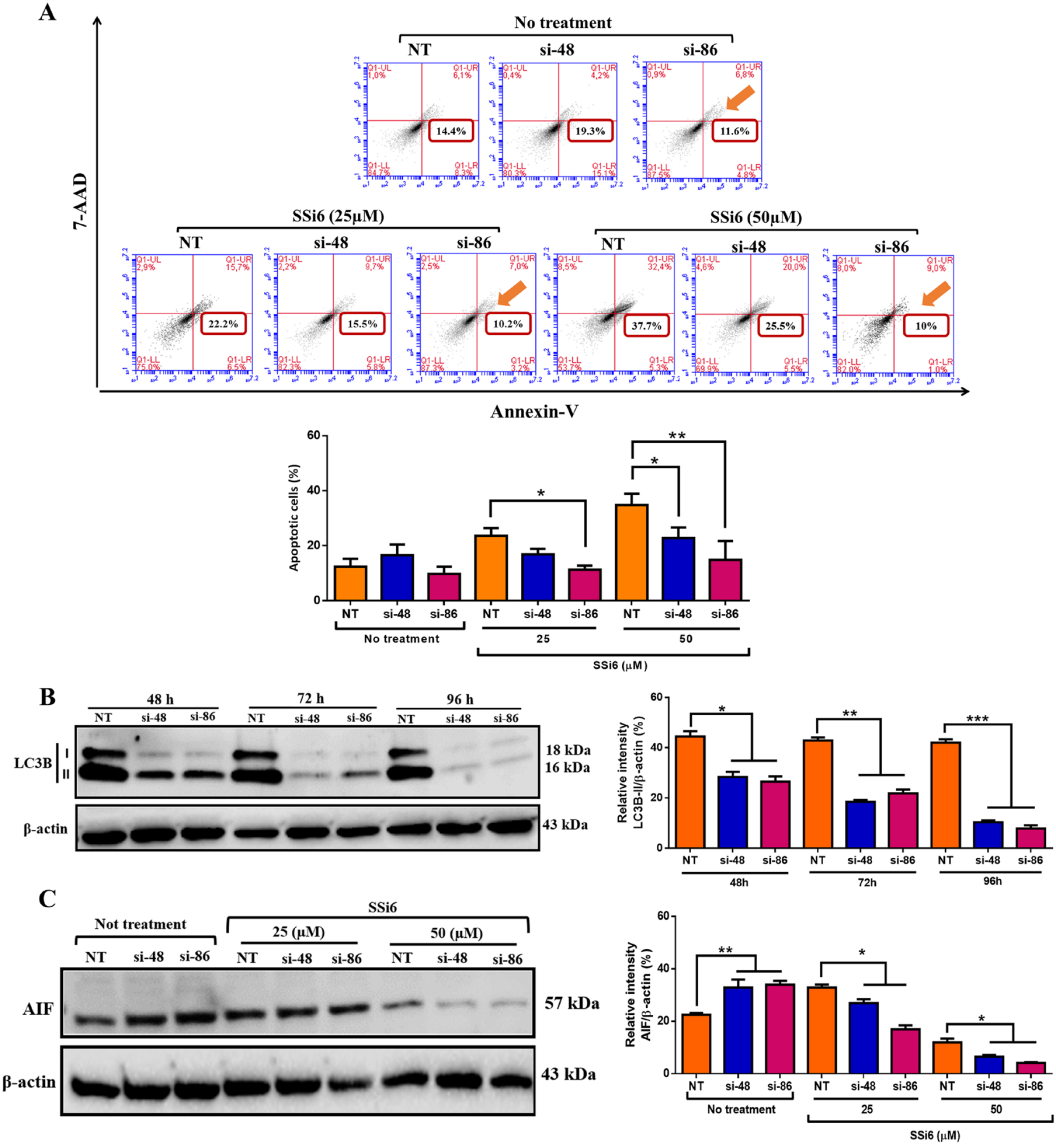
LC3B knockdown attenuates SSi6 apoptotic effects in TNBC cells **(A)** MDA-MB-231 cells were transfected with siRNA targeting LC3B or non-target siRNA (NT). (A) Suppression of autophagy partially inhibits apoptosis in MDA-MB-231 cells. Cells were treated with indicated concentrations of SSi6 for 24h, fixed and stained with PE-Annexin-V and 7-AAD. The si-86 was the siRNA with the higher effect on apoptosis (sum of the percentage of early and late apoptotic cells) and is represented by an orange arrow in histograms. Data represents mean ± SD of three independent assays in triplicate. Significance at the ^*^p<0.05, ^**^p<0.001 level using ANOVA followed by a Tukey's post-hoc analysis. NT: non-target control #1 siRNA; si-48: MAP1LC3B silencer siRNA-ID: s37748; si-86: MAP1LC3B silencer siRNA-ID: s224886. **(B)** LC3B (LC3B-I and LC3B-II) and **(C)** AIF expression was analyzed by western blotting after 48, 72, 96 and 14h respectively. β-actin was used as endogen control.

Finally, to demonstrate the importance of autophagy in the SSi6-induced caspase-independent apoptosis, LC3B in MDA-MB 231 cells were knocked down using siRNA. Was investigated whether LC3B knockdown would influence cell death upon SSi6 treatment. Indeed, LC3B silencing remarkably reduced SSi6-induced apoptosis in MDA-MB 231 cells (Figure 7A). LC3B-mediated autophagy is a well described mechanism in the literature. LC3B levels after 48, 72 and 96h of transfection demonstrated a silencing efficacy for both siRNA sequences used (si-48 and si-86) (Figure 7B). SSi6 treatment was unable to rescue the expression of LC3B protein in silenced cells (Supplementary Figure 5). Next it was analyzed whether the knockdown of the LC3B was associated with the reduction of AIF. The accumulation of free radicals upon SSi6 treatment induced partial loss of Δψm (see Figure 2C) leading to the release of apoptosis effector proteins located in the mitochondrial matrix, such as AIF, towards the cytoplasm. Furthermore, LC3B knockdown significantly reduced SSi6-mediated AIF expression in MDA-MB-231 cells (Figure 7C). Taken together, these data indicate that, at least to some extent, SSi6-induced apoptosis in TNBC cells strongly relies on prior autophagy events that were inhibited upon LC3B silencing.

## DISCUSSION

Clinical cancer therapies comprise physical treatment such as radiotherapy and surgery, as well as chemical treatment [37]. However, the outcome of these approaches is not always satisfactory, especially when tumor cells develop resistance to canonical apoptosis-inducing drugs. Some patients are not even sensitive to the existing chemotherapeutics [38]. Therefore, it is a matter of urgency to discover more pharmacologically active compounds, which can also be used as leading compounds for structural modifications in drug development.

The biological and pharmacological properties of some ginger derived compounds have already been well described in the literature. Gingerols (mainly 6G) are identified as the major constituents of plant rhizomes. In this study, 6G was chemically modified by conjugation to 2,4-DNPH in order to obtain a new semisynthetic compound, SSi6 [25]. There are no studies demonstrating the cytotoxic effects of this new substance, thus its effects were analyzed based on previous work that evaluated 6G cytotoxic and antitumor activities. These studies reported that 6G inhibited proliferation of A531 human squamous carcinoma (IC_50_ 300μM) [39], colon HCT-116 (IC_50_ 160.42μM) [40], hepatic HepG2 (IC_50_ 420μM) [41] and TBNC MDA-MB-231 cells (IC_50_ 684.3μM) [42]. In the present work, we demonstrated that the synthetic modification performed on 6G significantly increased its cytotoxicity against MDA-MB-21 cells approximately 17-fold, compared to the values shown for 6G.

Numerous studies report that apoptosis and autophagy may be concomitantly induced by antitumor compounds in order to kill tumor cells in a complex, coordinated and cooperative manner [43, 44]. Other studies have demonstrated that autophagy induction is an early event, detected within the first hours of treatment, with subsequent induction of apoptosis at longer incubation time [45]. Carnosol, a phenolic diterpene with reported antioxidant activity, induced autophagy within the first 3h of treatment, confirmed by the increase in LC3B protein levels, and subsequently caused apoptosis at longer treatment times, shown by PARP cleavage in MDA-MB-231 [46]. A recent study by Wong and co-workers [47] showed that 1,3-dibutyl-2-thiooxo-imidazolidine-4,5-dione (C1) triggers intracellular ROS production, simultaneously inducing autophagy and apoptosis in several types of tumor cells, including MDA-MB-231.

In agreement with these previous studies, our data showed that short-term incubation (2-6h of treatment) with SSi6 at 50 and 100μM induces autophagy in TNBC cells, which was initially observed by the occurrence of abundant cytoplasmic vesicles, characteristic of this type of cell death. In accordance with the hypothesis that SSi6 triggered autophagy, we then demonstrated that the vesicles observed upon SSi6 short-term treatment (2-6h) were AVOs, once again indicating autophagy occurrence. LC3B protein, a marker of autophagosome occurrence (and therefore autophagy), was also detected by immunostaining of SSi6-treated MDA-MB-231 cells at the same time points (2-6h). Apoptosis, accessed by flow cytometry, was not detected at 6h of SSi6 incubation but did occur at longer incubation times (24h). Accordingly, while after 1h of incubation SSi6 positively regulated LC3B gene expression and no changes were observed in *h*AIF (effector of apoptosis), upon 10h of SSi6 incubation, LC3B gene expression levels were back to normal and there was a significant upregulation on *h*AIF. These results corroborate the aforementioned literature and the hypothesis that SSi6 induces early autophagy that eventually culminates in apoptosis.

ROS accumulation increases oxidative damage, which leads to an increase in autophagy [20, 48] and causes a gradual loss of mitochondrial membrane polarization [45], which changes permeability properties and ultimately allows pro-apoptotic proteins, such as AIF, to be released into the cytoplasm. Apoptosis induction by SSi6 can be explained by the fact that as incubation time increases, the damage caused by autophagy and DNA fragmentation leads to the progression of cell death to apoptosis. In other words, when apoptosis became unrestrained, as observed after SSi6 treatment, not only autophagy will lead to cell death, but also the accumulation of ROS will induce partial/gradual mitochondrial membrane depolarization and AIF release, important features during caspase-independent cell death [49]. In the present study, SSi6 induced a significant increase in ROS generation, while pretreatment with NAC remarkably reversed the SSi6-induced inhibition of the autophagy and apoptosis in MDA-MB-231 cells.

Western blotting assays were performed to confirm the increase in apoptotic and autophagic protein levels in MDA-MB-231 cells exposed to SSi6. Alongside our results on gene expression, LC3B was upregulated upon 3 and 6h of SSi6 treatment, with AIF protein levels increasing at 14h of SSi6 treatment. Neither of these changes was observed in 6G and 2,4-DNPH-treated cells. These results are somehow consistent with what is described in the literature on 6G [42], although it was described that 6G promotes cell death in MDA-MB-231 cells by caspase-dependent apoptosis [50]. In particular, the 6G concentrations used to achieve such results were higher than the ones we tested [50], which could explain why it was not observed any changes in caspase-3 activation in 6G treated cells. Our results, however, demonstrated that SSi6 did not induce caspase-3 cleavage, the main effector protein in caspase-dependent apoptosis. Overall, these assays enable to demonstrate that SSi6 promotes apoptosis primarily through the induction of caspase-independent cell death, shown by a significant increase in AIF and a lack of cleaved caspase-3 protein expression.

Different studies in the literature suggest that autophagic flux plays an important role in pathological processes [51]. Autophagic flux is determined by the equilibrium between autophagosome formation and autophagosome clearance by lysosomes. Thus, defects in autophagy early or late steps cause autophagic dysfunction, which subsequently induces autophagic cell death. Xu *et al*. [52] found that physapubescin B (pB), a steroidal compound extracted from *Physalis pubescens*, combined with CQ depletion the autophagic flux in human colon carcinoma HCT116 cells, demonstrating that the anticancer potential of pB can be improved when combined with autophagy inhibitors. Moreover, the study of Qiu and colleagues [53] reported that tetrandrine (Tet:10μM), a natural product isolated from the root of *Stephania tetranda*, blocks autophagic flux in human prostate adenocarcinoma PC-3 and human renal adenocarcinoma 786-O cells, shown by the increase of LC3-II and SQSTM1/p62 when concomitantly treated with CQ (10μM) for 1h. Our results showed that SSi6 decrease autophagic flux when combined with CQ, causing an accumulation of LC3B-II, by inhibiting the degradation of autophagosomes, leading to subsequent induction of apoptotic cell death.

Wang *et al.* [54] found that berberine, an isoquinoline alkaloid derived from plants, decreased colony formation and induced caspase-independent cell death in the IMCE colon tumor cell line. The authors reported that the mechanism underlying berberine-induced cell death was through ROS-production-dependent AIF activation. Moreover, another study showed that dioscin, a saponin extracted from the roots of *Polygonatum zanlanscianense*, induced cell death via AIF-facilitating caspase-independent pathway, as well as down-regulation of anti-apoptotic proteins, such as Bcl-2 and cIAP-1 (Cellular Inhibitor of Apoptosis Protein 1) in breast cancer cells (MDA-MB-231, MDA-MB-453) [55]. Indeed, many polyphenols from natural sources have being reported to evoke autophagy and/or apoptosis in cancer cells. Concerning this, all results obtained in this work indicate that autophagy cell death is an important step that precedes apoptosis in the MDA-MB-231 cell line.

Finally, in order to elucidate whether SSi6-induced autophagy was a mandatory step for downstream apoptosis induction, we knocked down LC3B, one of the main proteins involved in autophagy, in MDA-MB 231 cells prior treatment with SSi6. Indeed, apoptosis occurrence, accessed by flow cytometry, was significantly decreased in siRNA-LC3B cells, compared to non-target control (siRNA-NT) cells upon SSi6 treatment. Accordingly, AIF protein levels did not increase (and were even downregulated) upon SSi6 treatment when LC3B was knocked down. However, autophagy, and consequently apoptosis, were not completely abrogated upon treatment with SSi6 when LC3B was knocked down, as other LC3 family members (mainly LC3A-II) can also be involved in autophagy [56]. These results strongly support the hypothesis that autophagy is a key initial step in SSi6-induced caspase-independent apoptosis. A previous study has also shown that 6G has the ability to induce autophagy and apoptosis in HeLa cells. In these cells, 6G induced several morphological changes, including phosphatidylserine externalization, DNA degradation, increased TUNEL positivity, PARP and caspase-3 expression and depolarization of mitochondrial membrane, showing evidence of mitochondrial-mediated apoptosis. On the other hand, death by autophagy induced by 6G was shown by fluorescence microscopy and flow cytometry at concentrations of 75, 100 and 125μg/ml after 24h of treatment [57]. Nevertheless, to the best of our knowledge, our research is the first to report a temporal and compulsory association between autophagy and apoptosis triggered by a ginger-derived substance in MDA-MB-231 cells.

In summary, this work reported that SSi6, a semisynthetic substance derived from 6G, increased ROS levels in human TNBC cells (MDA-MB-231), which triggered short-term autophagy and long-term caspase-independent apoptosis. The activation of these two mechanisms of cell death by SSi6 would be a promising strategy for breast cancer therapy, especially for cells that are resistant to canonical apoptosis-inducing agents, providing novel therapeutic tools and ultimately leading to new therapeutic strategies for cancer treatment.

## MATERIALS AND METHODS

### Reagents

SSi6 was obtained as described earlier [25] and dissolved in DMSO. We used the following reagents in our experiments: Acetone-2,4-dinitrophenylhydrazone (2,4-DNPH) was obtained by reaction between 2,4-dinitrophenylhydrazone and acetone, using the same methodology carried out to obtain of SSi6. ^1^HNMR analyses were performed for confirmation of their chemical structure. The final concentrations of Dimethyl sulfoxide (DMSO; Sigma-Aldrich, D4540) in the culture medium did not exceed 1%. The following reagents were used: DharmaFECT 4 (GE Healthcare, T-2004-01), acridine orange (catalog number A6014), rapamycin (catalog number R8781), wortmannin (catalog number W1628), propidium iodide (catalog number P4170), 3-(4,5-dimethylthiazol-2-yl)-2,5-diphenyltetrazolium bromide (MTT; catalog number M21281G), camptothecin (catalog number C9911), SYBR Green JumpStart Taq Ready Mix (catalog number S4438), RNaseA (catalog number R6513), 4′,6-Diamidine-2′-phenylindole dihydrochloride (DAPI; catalog number D9542), 2′,7′-Dichlorodihydrofluorescein diacetate (H_2_DCFDA; catalog number D6883), Hydrogen peroxide (H_2_O_2;_ catalog number H1009), Chloroquine (CQ; catalog number C6628), *N*-Acetyl-L-cysteine (NAC; catalog number A9165), CelLytic™ M (catalog number C2978) were purchased from Sigma-Aldrich. For western blot analysis, the following primary and secondary antibodies were used: anti-LC3B (Abcam, ab51520), anti-caspase-3 (Abcam, ab13847) and anti-AIF (Santa Cruz Biotechnology, sc-9416), HRP-conjugated goat rabbit secondary antibody (Abcam, ab6721). For immunostaining assay the anti-rabbit Alexa Fluor 488-conjugated antibody (Thermo Scientific, D12371) was used. The BD™ MitoScreen Kit and Apoptosis Detection Kit were obtained from BD Bioscience. The LC3B silencer siRNAs (s37748, s224886) and the non-target control (AM4611) were from Thermo Scientific.

### Cell culture

Non-malignant breast cells MCF-10A were cultured in Dulbecco's Modified Eagle Medium F12 (DMEM/F12; Thermo Scientific, 11320033) supplemented with 5% of horse serum (Sigma-Aldrich, H1270), 20ng/mL of Epidermal Growth Factor (EGF; Thermo Scientific, PHG0311), 0.5μg/mL of hydrocortisone (Sigma-Aldrich, H0888) 10μg/ml of insulin (Thermo Scientific, 12585-014) and 1% of penicillin/streptomycin (Vitrocell, 000403). TNBC MDA-MB-231 cells were cultured in DMEM supplemented with 10% of Fetal Bovine Serum (FBS) and appropriate antibiotics. Both cell lines were obtained from the Rio de Janeiro Cell Bank (BCRJ) and maintained at 37°C in an incubator with 95% of relative humidity and 5% CO_2_.

### Cytotoxicity assays

The effects of 6G, 2,4-DNPH and SSi6 on the cytotoxicity of MDA-MB-231 and MCF-10A cells were determined by colorimetric assays using MTT [3-(4,5-dimethylthiazol-2-yl)-2,5-diphenyltetrazolium bromide] [58]. Cells were seeded (1×10^4^ cells/100μL) into 96 well-plate (Greiner bio-one, 655180) and incubated until reaching 80% confluence. Afterwards, the cells were exposed to increasing concentrations of substances (3.12-100μM) for 48h. After treatment, the medium was removed, and cells were treated with MTT (1mg/mL) for 4h and formazan crystals were solubilized 100μL of DMSO. Absorbance was measured using an ELISA plate reader (spectrophotometer Labtech, LT-4000) at a wavelength of 540nm. Cytotoxicity assay was performed in comparison to the wells where the control cells (1% DMSO) were added instead of the tested compounds. Doxorubicin was used as a positive control of cell cytotoxicity [59].

### Clonogenic assays

MDA-MB-231 (3×10^2^/plate) were seeded into 6cm Petri dishes (Kasvi, K13-0060) and incubated at 37°C and 5% CO_2_ overnight (24h) and then treated with different concentrations (6.25, 12.5 and 15μM) of SSi6 and 6G for 48h. After this time, the culture medium was removed, and was replaced by complete culture medium (DMEM with 10% FBS) without treatment. The cells were maintained under the same conditions for a period of 10 days. After incubation, cells were fixed with methanol and acetic acid solution (3:1) for 5 min and stained with 0.5% crystal violet for 15 min. Colonies formed were analyzed in both number and size, using Image J software [28].

### Detection of acid vesicular organelles (AVOs)

MDA-MB-231 and MCF-10A (1×10^4^ cells/well) were seeded in a black 96 well-plate (Corning Costar, 3603) with a clear bottom and maintained at 37°C and 5% CO_2_ overnight. After incubation, cells were treated with SSi6 and 6G (25, 50 and 100μM) for 6h. After treatment, cells were washed with PBS and fixed with methanol for 10 min. Afterwards, cells were stained with acridine orange (AO; 1 μg/mL), which detects AVOs formation, for 15 min in the dark, then washed and stained with a solution of DAPI 1μg/mL (Life technologies, Carlsbad, CA), for nuclear staining, for 5min. Images were captured with automated high-resolution epifluorescence microscopy ImageXpress micro equipment (Molecular Devices, CA, USA) with a magnification of 400×. Rapamycin (500nM) and wortmannin (30μM) were used as a positive control for induction and inhibition of autophagy, respectively [28, 60].

### Immunostaining

To determine the presence of LC3B protein after SSi6 and 6G treatment in MDA-MB-231 and MCF-10A, cells were incubated with anti-LC3B primary antibody specific for LC3B-I proteins (cytoplasmic protein) and LC3B-II (anchored to autophagossome membrane) was used. MDA-MB-231 and MCF-10A (1×10^4^ cells/well) were seeded in a black 96 well-plate with a clear bottom (Corning Costar, 3603) and maintained at 37°C, 5% CO_2_ for 24h. Cells were exposed with 50μM of SSi6 and 100μM of 6G for 6h. Rapamycin (500nM) was used as the positive control of autophagy for 24h and for the negative control; cells were treated with 30μM wortmannin and added 50μM SSi6 for 6h of exposure. After treatment, the medium was removed, and cells were fixed with 4% paraformaldehyde (Sigma- Aldrich, P6148) for 20min and washed with PBS-glycine (50mM) (200μL) for 10min. After this, a block step was carried out for 1h with immunofluorescent solution (IF) + 10% bovine serum albumin (BSA; Sigma-Aldrich, A8412) in a buffer [(NaCl (1.30M), Na_2_HPO_4_ (0.13M), NaH_2_PO_4_ (0.029M), NaN_3_ (0.077M), Tween-20 (2% *v*/*v)* and Triton X-100 (0.5%), pH 7.4]. Subsequently, cells were incubated with anti-LC3B primary antibody (1:3000) for 3h at room temperature. Cells were washed three times with IF solution without BSA and incubated with a secondary antibody (Alexa Fluor^®^ 488-conjugated goat to rabbit IgG) for 1h and stained with DAPI 0.5μg/mL for 4min. Images were obtained using an automated microscope ImageXpress^®^ Micro XLS System (Molecular Devices) with a magnification of 400× and digital confocal adjust.

### Apoptosis detection by flow cytometry

Apoptotic activity of SSi6 on MDA-MB-231 was analyzed by flow cytometry using the PE-Annexin-V Apoptosis Detection Kit (BD Bioscience, 559763). Cells (1×10^5^ cells/well) were plated into 12 well-plates (Corning Costar, 3513) and after 24h of incubation at 37°C were exposed to different concentrations (25, 50 and 100μM) of SSi6 and 6G (100μM) during 6 and 14h. Cells were incubated with 5μL of PE-Annexin-V and 7-AAD for 15min in the dark. Analyses were performed in flow cytometer Accuri C6 (BD Bioscience, Franklin Lakes, NJ, USA) recording 10,000 events for each condition, after scraping the cells from the wells. Emitted fluorescence by each dye was quantified in CellQuest software (BD Bioscience, Franklin Lakes, NJ, USA), and it is proportional to the percentage of cells in apoptosis. The apoptotic rate of treatment was compared to the control (1% DMSO) and camptothecin (200μM) was used as the positive control of apoptosis [61].

### Reverse transcriptase quantitative real time PCR (RT-qPCR)

A reverse transcriptase (RT)-polymerase chain reaction (PCR) was performed to evaluate changes in the mRNA expression. MDA-MB-231 cells (1×10^6^/dish) were seeded into 6cm Petri dishes and after 24h of incubation, treated with SSi6 (25 and 50μM) and 6G (50 μM) for 1 and 10h. Total RNA was extracted using TRIzol reagent (Sigma-Aldrich, T9424). cDNAs were synthesized using the Enhanced Avian RT First Strand Synthesis kit (Sigma-Aldrich, STR1-1KT). Reactions were adjusted to each primer, depending on its melting temperature using Rotor Gene 6 software in Rotor-Gene RG 3000 equipment (Bio-Rad Laboratories, Hercules, CA, USA). In brief, 1μL of a reverse transcribed product template, 12.5μL of SYBR Green JumpStart Taq Ready Mix (Sigma-Aldrich, S4438) 10μL of pure water, 2μL of the primer pair and 0.5μL of cDNA at a final concentration 500nM made a final 25μL of the reaction system. The expression of genes related to apoptosis in treated cells was compared to control (1% DMSO). Primers used for amplification of MAP1LC3B, CASP3 and hAIF are described in Supplementary Table 1. For each gene, all samples were amplified simultaneously in duplicate in one assay run. Data represent three assays in duplicate and were normalized using the comparative cycle threshold (Ct) method. A blank with water, primers and SYBR Green instead of the template sample was performed. The target gene expression was normalized with endogen control Rpl37a, as previously described [62].

### Western blotting

MDA-MB-231 cells (1×10^6^/plate) were incubated for 24h with increasing concentrations of SSi6 (25, 50 and 100μM) and 6G (50 and 100μM) in Petri dishes (6cm) at 37°C. After incubation, cells were lysed using CelLytic™ M reagent (Sigma-Aldrich, St. Louis, MO, USA), according to the manufacturer's instructions in order to obtain the protein content. Protein concentrations of supernatants were determined using Pierce^®^ BCA Protein Assay kit (Thermo scientific, 23225). Protein samples (15μg) were applied to 4-20% mini-PROTEAN^®^ TGX™ Precast Gels (BioRad, 5871) for 1h at 100V, transferred to nitrocellulose membranes (BioRad, 1620145) and blocked with casein 1% (BioRad, 1610782) for 1h. All the processes followed the manufacturer's instructions. Next, the membranes were incubated overnight with anti-LC3B (1:3000), anti-Caspase-3 (1:1000) and anti-AIF (1:200) primary antibodies (Abcam, Cambrige, UK and Santa Cruz Biotechnology, Dallas, USA), followed by incubation with HRP-conjugated goat rabbit secondary antibody (1:5000) and for 1 h. β-actin was used as the endogenous control. Proteins were analyzed by chemiluminescence using Clarity™ Western ECL Substrate (BioRad, 1705060). Specific bands were visualized with a ChemiDoc MP imager (BioRad Laboratories, Hercules, CA, USA) and quantified with Image J software, normalized to β-actin antibody (1:3000) (Santa Cruz Biotechnology, sc-1616).

### Reactive oxygen species (ROS) production

MDA-MB-231 and MCF-10A cells (1.5×10^5^cells/well) were seeded in a 12-well plate and treated with different concentrations of SSi6 (25, 50 and 100μM) in the absence or presence of NAC (5mM) for 1 and 8h. After treatment, was added H_2_DCFDA [2′,7′-Dichlorodihydrofluorescein diacetate] (Sigma-Aldrich, D6883) was added at a concentration of 10μM for 30min before harvest. The fluorescence was measured using a Synergy H1 Hybrid Multi-Mode Microplate-Fluorimeter at a wavelength of λexcitation=400nm and λemission=525nm. ROS assay was performed compared to control cells (no treatment). Hydrogen peroxide (H_2_O_2;_ Sigma-Aldrich, H1009) was used as a positive control (200μM) of production intracellular ROS [63, 64].

### Mitochondrial membrane potential assay

The variation in mitochondrial membrane potential (ΔΨm) was investigated using BD™ MitoScreen Kit using the dye 5′.5′.6.6′- tetrachloro-1.1′.3.3′-tetraethylbenzimidazolcarbocyanine iodide (JC-1; BD Bioscience, 551302). MDA-MB-231 cells (1×10^5^cells/well) were seeded in a 12-well plate and treated with SSi6 (25, 50 and 100μM) for 8h of treatment. Afterwards, the mixture was incubated for 15 min with JC-1 (10μg/mL) in the dark. After incubation, the cells were washed twice with cold PBS, suspended in a total volume of 200μL and analyzed using a flow cytometer Accuri C6 (BD Bioscience, Franklin Lakes, NJ, USA) recording 10,000 events. During mitochondrial depolarization, JC-1 aggregates were dissociated to the monomer, which emitted green fluorescence in the cytoplasm during the decrease of mitochondrial membrane potential. Therefore, the changes of mitochondrial membrane potential were calculated as the ratio of red to green fluorescence [65]. Camptothecin at 200μM was used as the positive 521 control (8h incubation).

### Small interfering RNA (siRNA)

The silencer predesigned siRNAs for LC3B (s37748 and s224886) and the non-target control siRNA (AM4611) were transfected into the MDA-MB-231 cells using DharmaFECT 4 (GE Healthcare, T-2004-01), at 25nM final concentration, according to the manufacturer's instructions. The lyophilized siRNAs were ressuspended in sterile ultrapure water. After 72h of cell transfection, apoptosis and western blotting assays were performed, as described previously in the Material and Methods section.

### Statistical analysis

Each experiment was performed in triplicate and independently to guarantee the reliability and reproducibility of the results. Data were expressed as mean ± SD and statistical analyses were performed using one-way analysis of variance (ANOVA) (acceptable p level < 0.05), followed by Tukey's test, which compares all groups with control group. Analyses were performed using Microsoft Office Excel 2013 (Microsoft Corporation) and GraphPad Prism^®^ version 6.0 (GraphPad Software, San Diego, CA, USA) software.

## SUPPLEMENTARY MATERIALS FIGURES AND TABLE



## Abbreviations

6G: [6]-gingerol
7-AAD: 7-aminoactinomycin D
AIF: apoptosis-inducing factor
Acetone-2,4-DNFH: acetone-2,4-dinitrophenylhydrazine
AO: acridine orange
AVOs: Acid vesicle organelles
CASP3: caspase-3
cDNA: complementary deoxyribonucleic acid
CIA: caspase-independent apoptosis
CQ: chloroquine
DMSO: dimethyl sulfoxide
H_2_DCFDA: 2′,7′-dichlorodihydrofluorescein diacetate
H_2_O_2_: hydrogen peroxide
^1^HNMR: hydrogen nuclear magnetic resonance
IC_50_: 50% inhibitory concentration of cell viability
LC3B: protein associated with microtubules 1B light chain 3
mTOR: mechanistic target of rapamycin
MTT: [3-(4,5-dimethylthiazol-2-yl)-2
NAC: *N*-acetylcysteine
TNBC: triple-negative breast cancer
siRNA: small interfering RNA
ROS: reactive oxygen species
SD: standard deviation
