# Supplementary material for: Autophagy-dependent apoptosis is triggered by a semi-synthetic [6]-gingerol analogue in triple negative breast cancer cells
Source: Oncotarget. 2018 Jul 20;9(56):30787–804. doi: 10.18632/oncotarget.25704 (PMC6089392; doi:10.18632/oncotarget.25704)
Supplement: Supplementary file 1 [file oncotarget-09-30787-s001.pdf]

Autophagy-dependent apoptosis is triggered by a semi-synthetic [6]-gingerol analogue in triple negative breast cancer cells

SUPPLEMENTARY MATERIALS

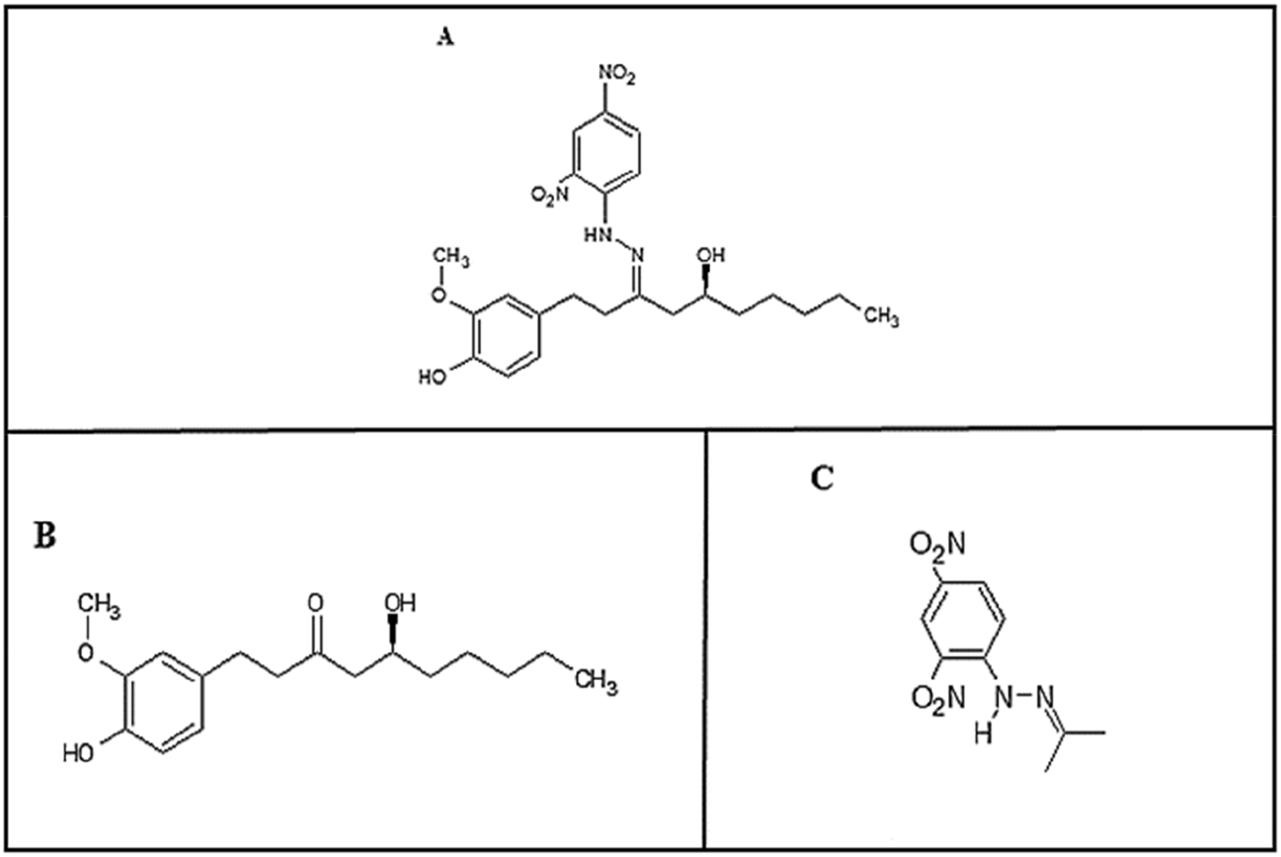

Supplementary Figure 1: Molecular structure of the substances. **(A)** SSi6 **(B)** [6]-gingerol (6G), **(C)** Acetone-2,4-dinitrophenylhydrazone (2,4-DNPH).

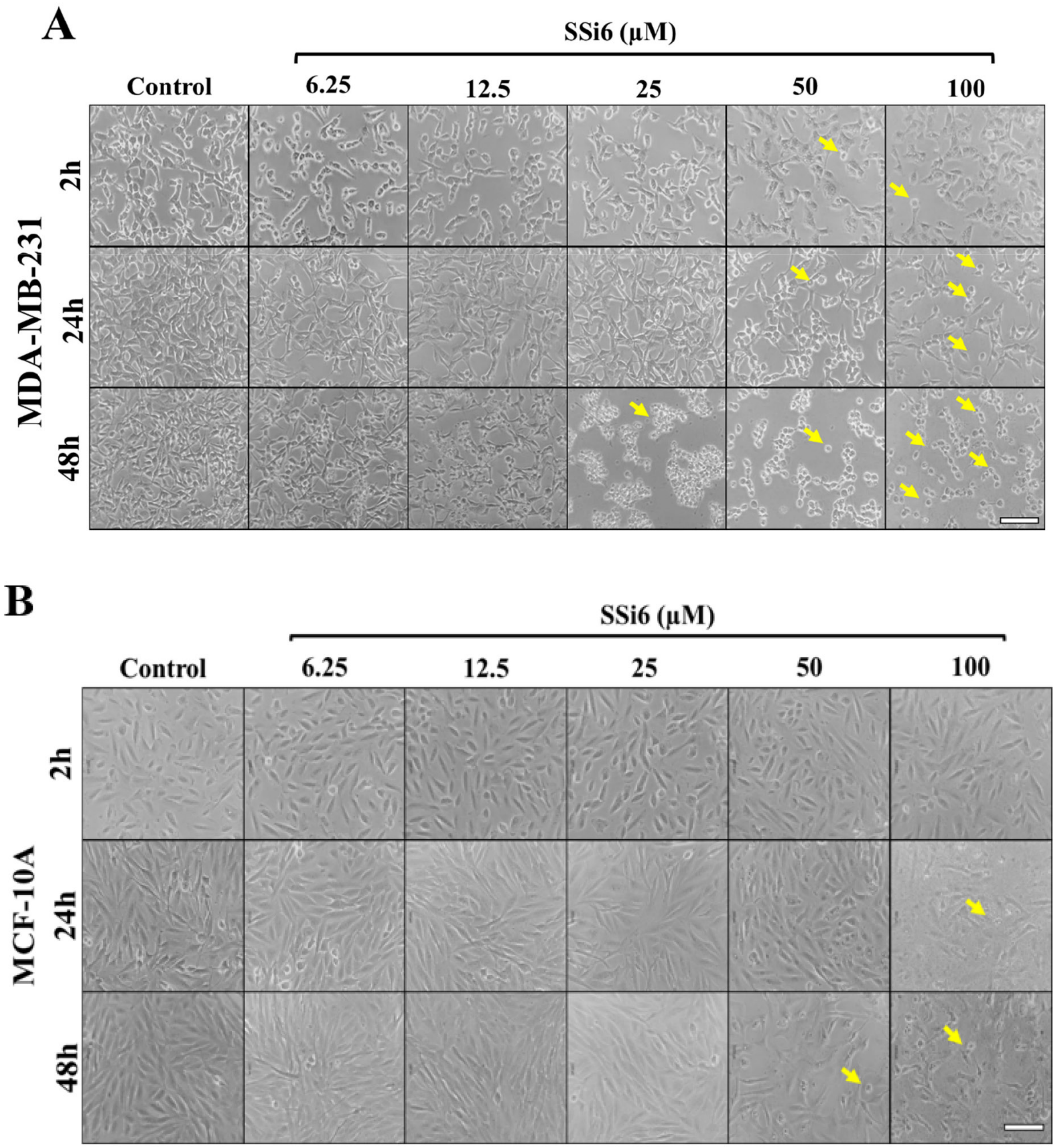

Supplementary Figure 2: Effects of SSi6 on cell morphology. **(A)** MDA-MB-231 and **(B)** MCF-10A ($1x10^5$) cells were treated with indicated concentrations of SSi6. Higher concentrations and time incubations with SSi6 induce round morphology cell detachment (yellow arrows). Images were captured after 2 and 24h under an inverted microscope at amplification of 100×, scale bar= 100 µm.

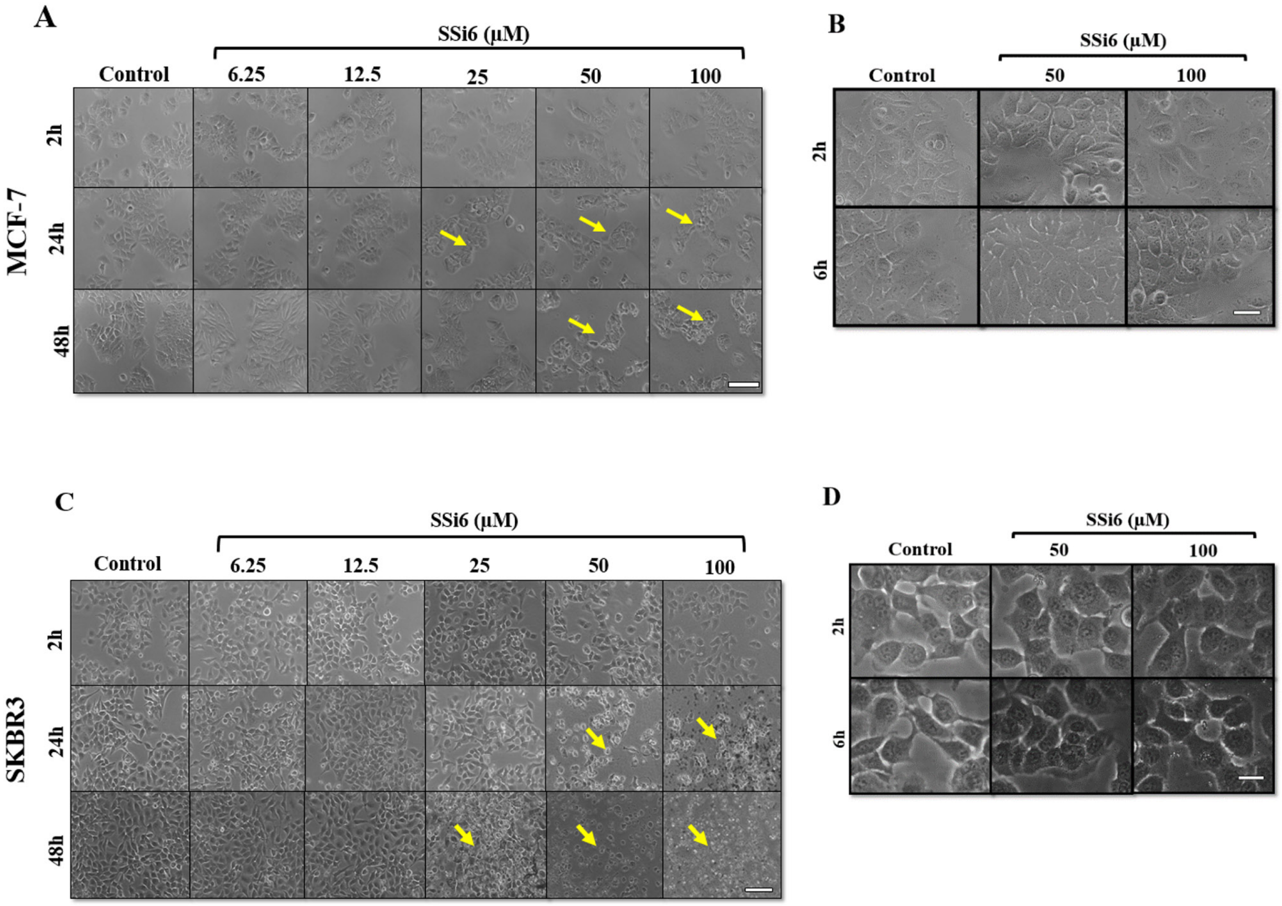

Supplementary Figure 3: Effects of SSi6 on the cell morphology of non-triple negative breast cancer cells. (**A** and **B**) MCF-7 and (**C** and **D**) SKBR3 cells were treated with SSi6 (yellow arrows indicate changes in cell morphology). Images were captured at various times of treatment, under an inverted microscope at amplification of 100 and 200×, scale bar= 100 and 50 μm respectively.

A

IC_{50} ± SD (µM)		
**Substance**	**24h**	**48h**
SSi6	>100	35.56 ± 0.73

B

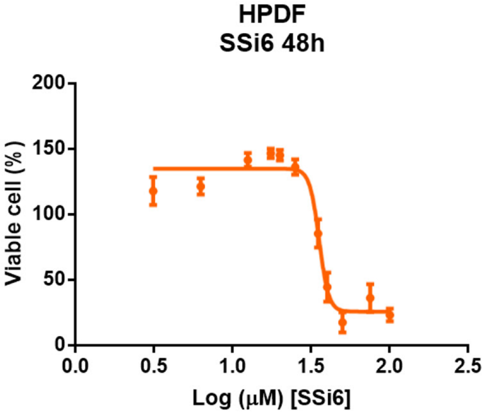

C **D**

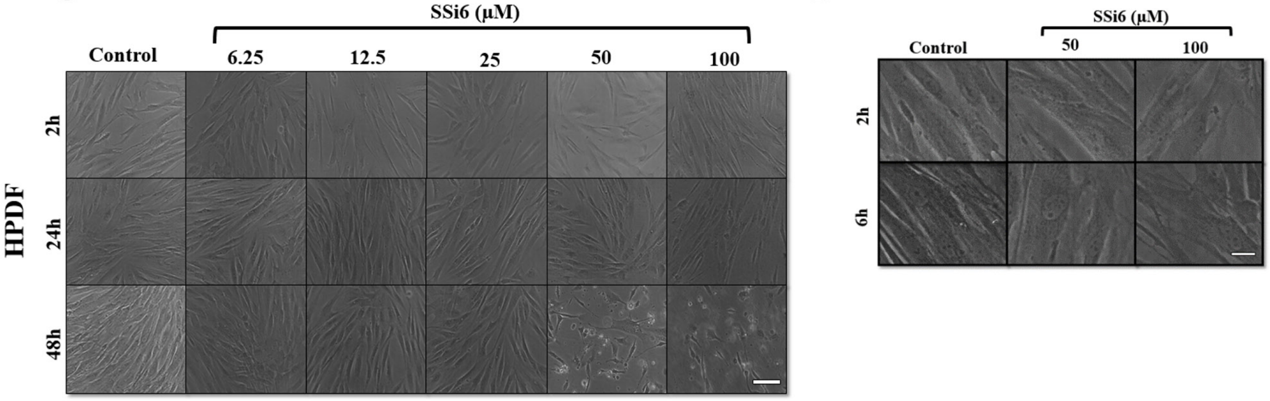

Supplementary Figure 4: Effects of SSi6 on the cytotoxicity and morphology assay in Human Primary Dermal Fibroblast cells (HPDF). **(A)** IC_{50} values of 24 and 48h treatment of SSi6 **(B)** IC_{50} plot on HPDF cells treated with SSi6 for 48h. Values are expressed as mean ± SD of three independent assays in triplicate. **(C)** HPDF cells were treated with SSi6 for 2, 24 and 48h (amplification 100×, scale bar=100 µm). **(D)** Cells were treated with substance for 2 and 6h, examined using an inverted microscope (amplification 200×), scale bar =50µm.

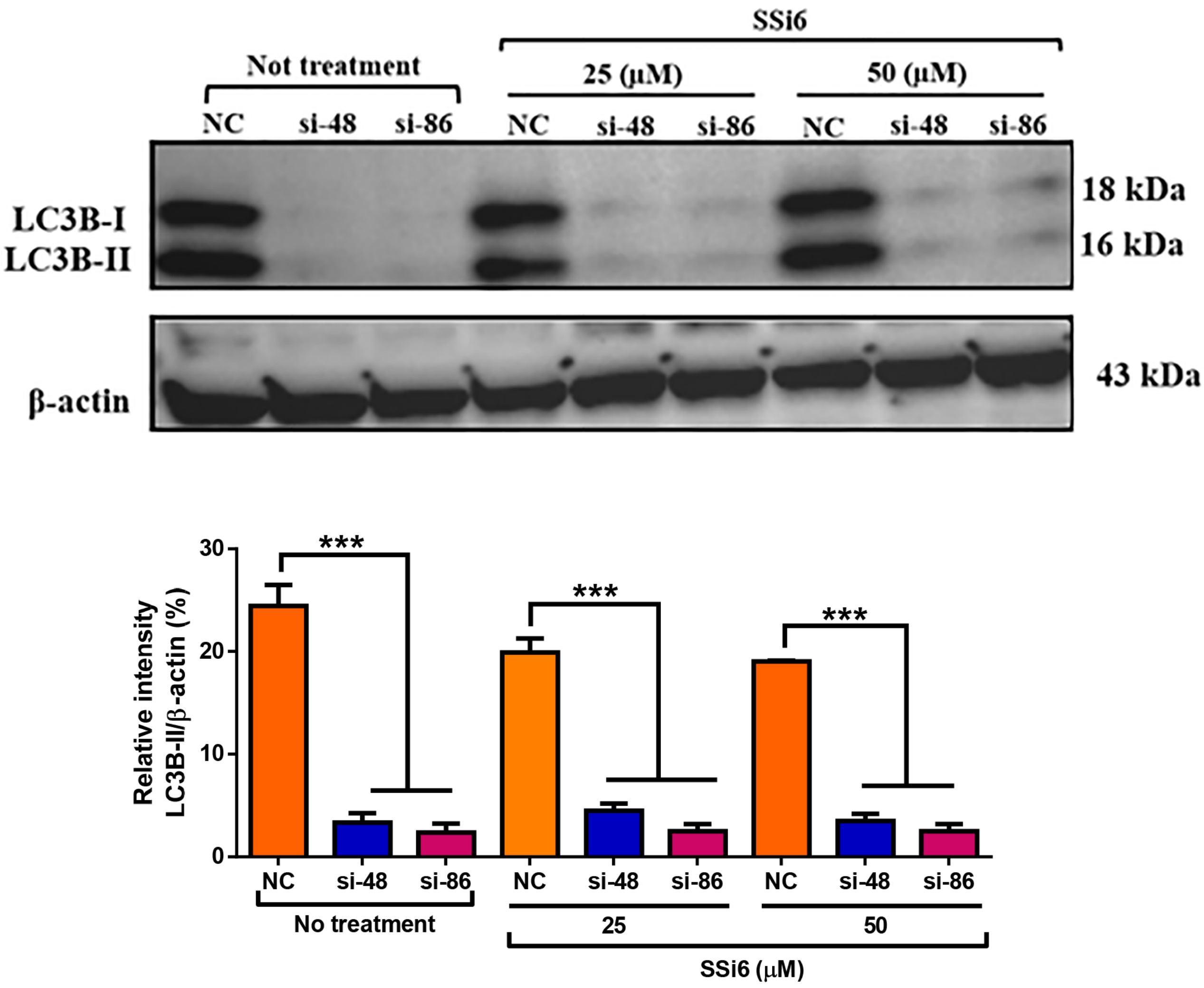

Supplementary Figure 5: Inhibition of autophagy in MDA-MB-231. Cells were transfected with siRNA targeting LC3B and treated at the indicated concentrations of SSi6. Cells were incubated (72h) with scrambled and LC3B-targeting siRNA. Silenced MDA-MB-231 was seeded in 6-well plates and analyzed by western blotting as described in Material and Methods.

Supplementary Table 1: List of primers used in the quantitative real-time RT-PCR analysis

Gene Symbol	Gene name	Accession number	Sequences (primer forward- primer reverse)
MAP1LC3B	Microtubule-associated proteins 1A/1B light chain 3B	NC_000016.10	5’GAG CAG CAT CCA ACC AAA A3’
			5’CCA TGC TGT GTC CGT TCA3’
CASP3	Caspase-3	NM_004346.3	5’TCC TAG CGG ATG GGT GCT AT3’
			5’ CGA GCA CTC ACG AAA CTC TTC3’
hAIF	Apoptosis-inducing factor	NC_000023.11	5’AAG TCA GAC GAG AGG GGG TTA3’
			5’ GCC AAC TCA ACA TTG GGC T3’
Rpl37a	Ribosomal protein L37a	NC_000002.12	5’GCC AGC ACG CCA AGT ACAC’3
			5’CCC CAC AGC TCG TCT CTT CA3’
